# Caregiver acceptance of malaria vaccination for children under 5 years of age and associated factors: cross-sectional household survey, Guinea and Sierra Leone, 2022

**DOI:** 10.1186/s12936-023-04783-0

**Published:** 2023-11-20

**Authors:** Klara Röbl, Hanna-Tina Fischer, Alexandre Delamou, Abdul Karim Mbawah, Brogan Geurts, Lukas Feddern, Habibata Baldé, Ibrahima Kaba, Francisco Pozo-Martin, Heide Weishaar, Sara Menelik-Obbarius, Gerrit Burger, Viorela Diaconu, Achim Dörre, Charbel El Bcheraoui

**Affiliations:** 1 Department of Infectious Disease Epidemiology, Robert Koch-Institute, Seestraße 10, 13353 Berlin, Germany; 2 Postgraduate Training for Applied Epidemiology (PAE), Robert Koch-Institute, Seestraße 10, 13353 Berlin, Germany; 3 ECDC Fellowship Programme, Field Epidemiology Path (EPIET), European Centre for Disease Prevention and Control (ECDC), Gustav III:S Boulevard 40, 16973 Solna, Sweden; 4 Evidence-Based Public Health, Centre for International Health Protection, Robert Koch-Institute, Nordufer 20, 13353 Berlin, Germany; 5 Centre d’Excellence Africain pour la Prévention et le Contrôle des Maladies Transmissibles (CEA-PCMT), Université Gamal Abdel Nasser de Conakry (UGANC), Dixinn, PoBox 1017, Conakry, Guinea; 6 Faculté des sciences techniques de la santé (FSTS), Université Gamal Abdel Nasser de Conakry (UGANC), Conakry, Guinea; 7 College of Medicine and Allied Health Sciences (COMAHS), University of Sierra Leone, Connaught Hospital, Freetown, Sierra Leone; 8 Charité-Universitätsmedizin Berlin, Charitéplatz 1, 10117 Berlin, Germany

**Keywords:** Malaria vaccines, Child, Caregivers, Guinea, Sierra Leone, Cross-sectional studies, Surveys and questionnaires, Logistic models, Immunization programs, Lot quality assurance sampling

## Abstract

**Background:**

Malaria is a leading cause of death and reduced life span in Guinea and Sierra Leone, where plans for rolling out the malaria vaccine for children are being made. There is little evidence about caregiver acceptance rates to guide roll-out policies. To inform future vaccine implementation planning, this analysis aimed to assess potential malaria vaccine acceptance by caregivers and identify factors associated with acceptance in Guinea and Sierra Leone.

**Methods:**

A cross-sectional household survey using lot quality assurance sampling was conducted in three regions per country between May 2022 and August 2022. The first survey respondent in each household provided sociodemographic information. A household member responsible for childcare shared their likelihood of accepting a malaria vaccine for their children under 5 years and details about children’s health. The prevalence of caregiver vaccine acceptance was calculated and associated factors were explored using multivariable logistic regression modelling calculating adjusted odds ratios (aOR) with 95% confidence intervals (CI).

**Results:**

Caregivers in 76% of 702 sampled households in Guinea and 81% of 575 households in Sierra Leone were accepting of a potential vaccine for their children. In both countries, acceptance was lower in remote areas than in urban areas (Guinea: aOR 0.22 [95%CI 0.09–0.50], Sierra Leone: 0.17 [0.06–0.47]). In Guinea, acceptance was lower among caregivers living in the richest households compared to the poorest households (0.10 [0.04–0.24]), among those whose children were tested for malaria when febrile (0.54 [0.34–0.85]) and in households adopting more preventative measures against malaria (0.39 [0.25–0.62]). Better knowledge of the cause of malaria infection was associated with increased acceptance (3.46 [1.01–11.87]). In Sierra Leone, vaccine acceptance was higher among caregivers living in households where the first respondent had higher levels of education as compared to lower levels (2.32 [1.05–5.11]).

**Conclusion:**

In both countries, malaria vaccine acceptance seems promising for future vaccine roll-out programmes. Policy makers might consider regional differences, sociodemographic factors, and levels of knowledge about malaria for optimization of implementation strategies. Raising awareness about the benefits of comprehensive malaria control efforts, including vaccination and other preventive measures, requires attention in upcoming campaigns.

**Supplementary Information:**

The online version contains supplementary material available at 10.1186/s12936-023-04783-0.

## Background

Malaria accounts for a substantial proportion of disease burden worldwide, and disproportionally affects children [1]. In 2021, an estimated 247 million cases and 619,000 deaths due to malaria occurred globally, of which 95% and 96%, respectively affected the World Health Organization (WHO) African region [2]. Of these deaths, about 79% were children below the age of 5 years, making them particularly vulnerable [2].

In both Guinea and Sierra Leone, malaria is endemic and the whole population is at risk, with an estimated incidence of about 331/1000 and 329/1000, respectively in 2021; this meets the WHO criteria for moderate transmission settings [2–5]. The parasite *Plasmodium falciparum* accounts for almost all malaria cases [2]. In both countries, malaria was the second leading cause of death for children under 5 years in 2019, accounting for 18.1% of all deaths in that age group in Guinea and for 22.9% in Sierra Leone [6]. The latest malaria indicator surveys conducted in both countries reported an estimated malaria prevalence according to microscopic diagnostics of 17% among children aged 6 to 59 months in Guinea and 22% in Sierra Leone [7].

Malaria control strategies must incorporate a set of measures based on prevention and case management, rather than relying on any single intervention [8]. In 2015, a new preventive measure for malaria control became available, when the first vaccine ever against *P. falciparum* malaria, RTS,S/AS01, received a positive assessment by a regulatory authority (the European Medicines Agency) for use in children aged 6 weeks to 17 months [9, 10]. Subsequently, a pilot roll-out program in three sub-Saharan African countries was launched to assess the vaccine’s effectiveness under real-world conditions [11]. An interim assessment of the impact conducted 24 months after the launch of the roll-out revealed a 30% reduction in hospital admissions with severe malaria in implementation areas [12]. Consequently, in 2021, the WHO recommended the first vaccine against *P. falciparum* malaria for wide use in children aged 5 months and above in moderate to high transmission settings complementing comprehensive malaria control programs [13]. Making malaria vaccination an integral part of malaria control efforts is hoped to increase access to preventive measures for children, thus reducing inequities and the overall burden of the disease in children [8, 14].

However, the benefits of vaccination as part of malaria control efforts depends on vaccine acceptance and subsequent uptake [15]. The WHO considers vaccine hesitancy a substantial challenge for global health, which has recently been on the rise in low- and middle-income countries (LMIC) [16, 17]. Strategies for vaccine implementation should, therefore, be informed by evidence about people’s attitudes towards vaccination to optimize uptake [18, 19]. A systematic review by Dimala et al*.* [20] indicated that malaria vaccine acceptance strongly depends on the socio-cultural context. Therefore, context- and country-specific evidence is needed to develop successful vaccine implementation policies. Ideally, information is gathered in advance to effectively engage communities at an early stage during roll-out, as this was identified as a weak spot in pilot programmes [21].

The roll-out of the malaria vaccine is planned for 2024 in both Guinea and Sierra Leone, but survey evidence for potential caregiver acceptance of malaria vaccines for children in these countries is lacking. To inform the Expanded Program on Immunization in both countries, the aim of this study was to (i) assess potential malaria vaccine acceptance by caregivers of children under 5 years, (ii) identify factors associated with vaccine acceptance to help plan upcoming vaccine introduction campaigns, and (iii) generate hypotheses for further research.

## Methods

Cross-sectional household surveys were conducted in Guinea and Sierra Leone within the framework of a mixed methodology research project called “Assessing the effect of the COVID-19 pandemic on health systems in Guinea and Sierra Leone (ACGSL): the case of malaria”, with the aim of assessing the resilience of their national health systems amid the COVID-19 pandemic. Ethics approval for the study was received by the Ethics Committee of the Ärztekammer Berlin in Germany (Eth-76/21), the Sierra Leone Ethics and Scientific Review Committee (15 March 2022), and the Comité National d'Ethique pour la Recherche en Santé (CNERS) in Guinea (013/CNERS7/21).

### Sampling and study area

**Fig. 1:**
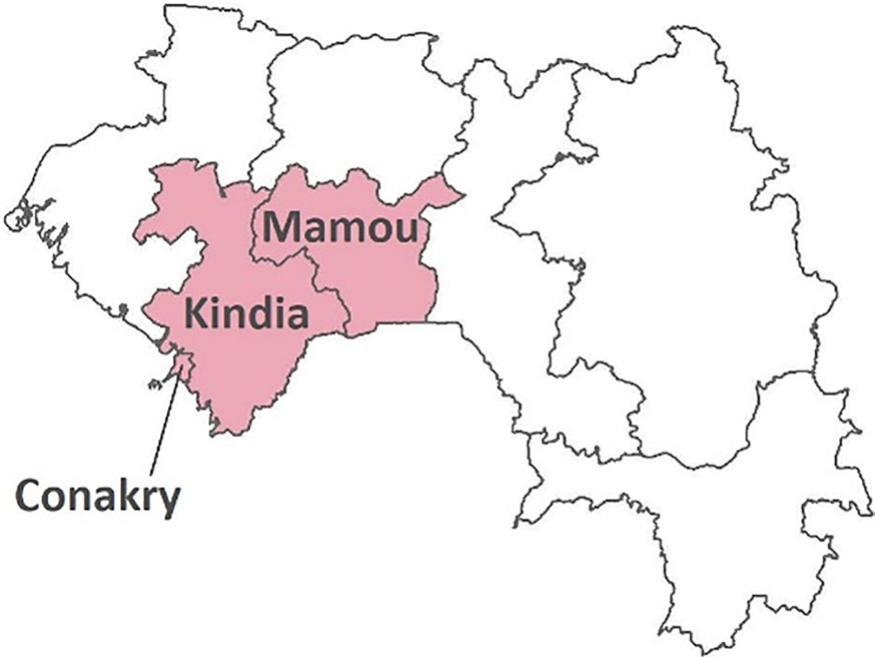
Selected regions in Guinea

**Fig. 2:**
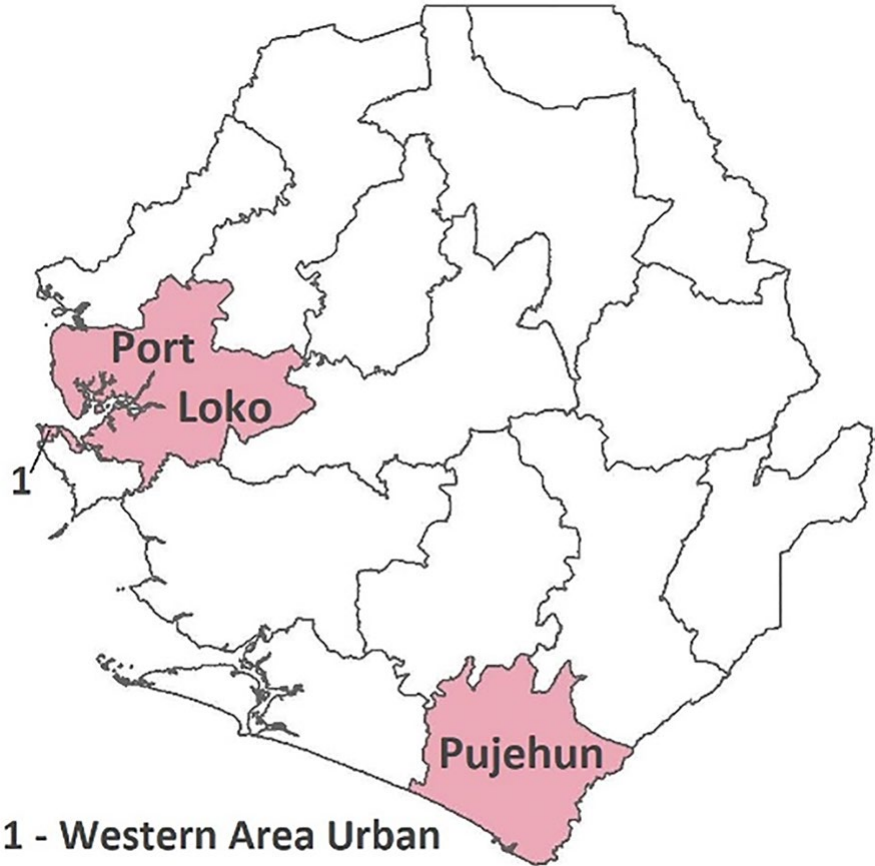
Selected districts in Sierra Leone

The sampling of households followed a lot quality assurance sampling (LQAS) approach based on a representative sample of health facilities. Three administrative regions in Guinea and three districts in Sierra Leone were purposively identified based on their relative burden of malaria and COVID-19, in order to include areas with high, medium and low burden of both diseases. In each country one urban area (Conakry in Guinea and Western Area Urban in Sierra Leone) and two rural ones (Kindia, Mamou in Guinea, and Port Loko, Pujehun in Sierra Leone) were selected (Figs. 1, 2).

In 2021, the estimated prevalence of malaria in children aged 6–59 months diagnosed by microscopy in the regions studied in Guinea ranged from 0.4% in Conakry to 13.2% in Mamou and 18.2% in Kindia [7]. Of the selected districts in Sierra Leone, the estimated prevalence of malaria in children aged 6 to 59 months in 2021 was 7.5% in the Western Area Urban to 22.9% in Port Loko and 25.8% in Pujehun [10].

Within the selected regions/districts, a systematic random procedure was used to identify 90 health facilities in Guinea and 67 in Sierra Leone. Each health facility serves a defined catchment area of households. Following the LQAS method, 19 households per catchment area were identified in Guinea and 20 households per catchment area in Sierra Leone for inclusion in the household survey. These households were randomly selected from two strata within the catchment area according to their respective proximity to the health facility: one community located in close proximity to the health facility and one located further away.

### Survey structure

Household survey respondents provided their informed consent, at least verbally prior to participation in the study, which was recorded in the survey software ODK. The household questionnaire included distinct modules directed to different respondents based on their caregiving relationship in the household. Initially, in each surveyed household, a first respondent provided information about the household wealth, socio-demographic factors, their educational level, and their trust in the healthcare system. If a child under 5 years lived in the household who had been ill with fever within 30 days prior to the survey, the child’s caregiver was sought to be interviewed, if they were available and consented to participate. Each caregiver provided information about their child’s health with a focus on malaria prevention, testing, and treatment of the child, as well as the caregiver’s knowledge about malaria and potential malaria vaccine acceptance. Respondents were asked to indicate whether they were very unlikely, unlikely, neither likely nor unlikely, likely or very likely in favour of malaria vaccination for their child if there was an effective vaccine to prevent malaria in children under five.

### Analysis

The R software (version 4.1.3.0, R Foundation for Statistical Computing, Vienna, Austria) was used for data analysis. Caregiver vaccine acceptance levels were analysed descriptively in both countries. Given that future interventions to increase vaccine acceptance might specifically target those who are opposed or indecisive, caregivers who stated their willingness to be either very unlikely, unlikely, or neither unlikely nor likely were compared to those who were likely or very likely in favour of vaccination. Therefore, vaccine acceptance was recoded into a binary outcome variable.

### Variable selection

A literature search was performed to identify factors associated with malaria vaccine acceptance. This served as a basis for the selection of variables to be included in the regression models.

Religious beliefs, educational levels, farming occupation, household wealth, the number of children under 5 years residing in the household, and the area of residence seem to influence malaria vaccine acceptance [15, 19, 22–25]. Studies from sub-Saharan African countries report both increased and decreased malaria vaccine acceptance with higher caregiver age [22, 23]. Possible side effects of vaccination, knowledge and awareness about the vaccine, previous experience with childhood vaccinations, the perception of there being too many childhood vaccinations, the need for multiple injections, and associated costs were reported to influence malaria vaccine acceptance [15, 22, 24–27]. Additionally, the perceived risk of malaria and the availability of other preventive measures against the disease seem to be associated with vaccine acceptance [15, 28]. One study found the encounter of messages about malaria within the last six months to be associated with vaccine awareness [22], which might increase risk perception similarly to knowledge about the disease [29–31]. Assuming that malaria testing behaviour is associated with increased risk perception, the former might also be associated with vaccine acceptance [29].

Satisfaction with health care services was reported to be associated with malaria vaccine acceptance [23]. In this study, no information about satisfaction was collected, but self-reported trust in the health care system was used as a proxy. The variable was collected on a five-point Likert scale and recoded into a binary scale (Additional file 1).

Based on these findings, and considering that the questionnaire which was used in this study did not cover vaccine-related factors possibly influencing acceptance, nor caregiver age or gender, the following variables were included in the analysis, most of which were collected at the household level:

- Region/district of residence.
- Household wealth index.
- First respondent’s self-reported educational level.
- Self-reported farming occupation.
- Self-reported number of children under 5 years living in the household.
- Self-reported number of preventive measures taken against malaria in household.
- First respondent’s self-reported trust in the healthcare system.
- Self-reported exposure to messages or advertisement about malaria within the six months prior to the survey.
- Caregiver’s ability to correctly identify mosquito bites as the cause of infection with malaria.
- Self-reported testing for malaria during child’s febrile illness.

An approach suggested by the World Food Programme was adapted to calculate the wealth index as a single measure of a household’s living standard based on the following variables: household size, ownership of assets (e.g. internet, radio), materials used for housing construction, type of water access and sanitation facilities [32]. The correlation between variables was assessed and those poorly correlated to most other variables were excluded (Pearson’s *r* < 0.1). The suitability of the selected variables was checked by calculating overall Kaiser–Meyer–Olkin measures (≥ 0.6) and Bartlett’s test of sphericity (p < 0.05). Principal component analysis was used to generate a normalized score, which was then divided into quintiles, whereby a higher score indicates increasing wealth.

Pearson’s chi-squared tests and Fisher’s exact tests were used to assess potential univariable associations between the explanatory variables and vaccine acceptance. Exploratory multivariable logistic regression was then conducted calculating odds ratios with 95% confidence intervals for both countries separately, in order to identify factors which are associated with malaria vaccine acceptance among caregivers [33]. Stepwise selection (both forward and backward) of variables was performed based on the Akaike Information Criterion (AIC) to obtain the final models [34–36]. *P*-values smaller than 0.05 were considered significant (Additional file 2).

Validation and model diagnostics included calculation of model accuracy, analysis of goodness of fit by conducting likelihood ratio tests, Hosmer–Lemeshow tests and assessing McFadden’s pseudo *R*^2^, assessment of multicollinearity by examining variance inflation factors, assessment of specification error and influential values, and testing for interactions [37, 38] (Additional file 3). Sensitivity analysis involved comparing the variable estimates from the final model to models including a greater number of variables (Additional file 2).

## Results

Between May and August 2022, 1710 households in Guinea and 1331 households in Sierra Leone participated in the survey. Within these households, 717 (42%) in Guinea and 576 (43%) in Sierra Leone had a child under 5 years who had been ill with fever within the last 30 days. Of those, 702 (97.9%) households in Guinea and 575 (99.8%) in Sierra Leone had a caregiver available who consented to participate in the study. Results of this analysis only refer to households with recently febrile children and consenting caregivers.

### Guinea

Most caregivers with children under 5 years old who had recently been ill with fever lived in the rural regions of Kindia (55%, *n* = 388) and Mamou (36%, *n* = 250), and only a smaller proportion in the country’s capital Conakry (9%, *n* = 64). In the majority of households with consenting caregivers (54%, *n* = 382), the first respondent reported not to have any formal education. 309 (44%) of first respondents indicated their main occupation to be farming (Table 1).

**Table 1:** Guinea: description of study population and vaccine acceptance, results of univariable and multivariable analysis

Characteristics	Households where caregivers consented *N* = 702	Multivariable logistic regression, stepwise selected model based on AIC^a^ *N* = 655^b^ AIC = 581.12, Pseudo-R^2^ (McFadden) = 0.24		
	N (%)	N willing to vaccinate/N total (%)	aOR (95% CI)	p-value
Region (*χ*^2^ = 16.91, *p* < 0.001)^e^				
Conakry	64 (9)	42/63 (67)	–	–
Kindia	388 (55)	288/355 (81)	0.60 (0.27, 1.33)	0.207
Mamou	250 (36)	165/237 (70)	0.22 (0.09, 0.50)	** < 0.001**
Household wealth index (*χ*^2^ = 60.27, *p* < 0.001)^e^				
1st quintile	151 (22)	123/136 (90)	–	–
2nd quintile	171 (24)	129/162 (80)	0.49 (0.23, 1.04)	0.064
3rd quintile	137 (20)	104/127 (82)	0.87 (0.39, 1.94)	0.731
4th quintile	128 (18)	84/119 (71)	0.34 (0.16, 0.74)	**0.006**
5th quintile	115 (16)	55/111 (50)	0.10 (0.04, 0.24)	** < 0.001**
Farming occupation^c^ (*χ*^2^ = 4.79, *p* = 0.029)^e^				
No	393 (56)			
Yes	309 (44)			
Education^c^ (*χ*^2^ = 10.72, *p* = 0.005)^e^				
No formal education	382 (54)			
Informal or Koranic school	60 (9)			
Primary school or higher	260 (37)			
Number of children < 5 years in household (*χ*^2^ = 0.72, *p* = 0.70)^e^				
1	265 (38)			
2	229 (33)			
More than 2	208 (29)			
Number of preventive measures taken against malaria in household (*χ*^2^ = 52.84, *p* < 0.001)^e^				
0–3	398 (57)	316/363 (87)	–	–
4 or more	304 (43)	179/292 (61)	0.39 (0.25, 0.62)	** < 0.001**
Trust in healthcare system^c^ (Fisher’s exact test, *p* = 0.38)^e^				
No or rather no trust	16 (2)	14/16 (88)	–	–
Some or a lot of trust	643 (92)	481/649 (75)	0.29 (0.06, 1.44)	0.131
Missing or neutral	43 (6)			
Messages or advertisement about malaria seen during past 6 months^c^ (*χ*^2^ = 0.03, *p* = 0.87)^e^				
No	116 (17)			
Yes	584 (83)			
Missing	2 (0)			
Caregiver can identify the cause of malaria infection (*χ*^2^ = 67.35, *p* < 0.001)^e^				
No	45 (6)	35/39 (90)	–	–
Partially	416 (60)	251/393 (64)	0.56 (0.18, 1.74)	0.319
Yes	241 (34)	209/223 (94)	3.46 (1.01, 11.87)	**0.048**
Malaria test was performed during child’s febrile illness (*χ*^2^ = 5.21, *p* = 0.023)^e^				
No	268 (38)	199/246 (81)	–	–
Yes	432 (62)	296/409 (72)	0.54 (0.34, 0.85)	**0.007**
Missing	2 (0)			
Willingness to vaccinate^d^				
Rather unlikely or undecided	170 (24)			
Rather likely	532 (76)			
Missing	0			

Bolded p-values are considered to be significant

^a^Akaike information criterion

^b^Excluding missing data

^c^Of first respondent

^d^Dependent variable

^e^Result of univariable analysis

#### Malaria vaccine acceptance

532 (76%) caregivers reported that they would likely or very likely accept a vaccine against malaria for their child if it was available. 170 (24%) caregivers stated that they would be either unlikely or very unlikely to accept a vaccine for their child or that they were indecisive (Table 1).

#### Factors associated with vaccine acceptance

Results of the multivariable analysis to identify factors associated with malaria vaccine acceptance among caregivers in Guinea are displayed in Table 1. A significant association between the area of residence and vaccine acceptance was found. In Mamou, the most distant region from the capital, the odds of vaccine acceptance were significantly lower (aOR: 0.22; 95% CI: 0.09–0.50) compared to the capital Conakry. Further, significantly lower odds of vaccine acceptance were found in the 5th (aOR: 0.10; 95% CI: 0.04–0.24) and 4th (aOR: 0.34; 95% CI: 0.16–0.74) wealth index quintile, and lower odds of borderline significance were found in the 2nd (aOR: 0.49, 95% CI: 0.23–1.04) wealth index quintile when compared to the 1st quintile, i.e. the poorest households.

Among caregivers who live in households that reported they apply four or more measures against malaria, the odds of vaccine acceptance were significantly lower (aOR: 0.39; 95% CI: 0.25–0.62) compared to those who reported to have up to three measures in place. Similarly, caregivers who reported that their febrile children were tested for malaria had significantly lower odds (aOR: 0.54; 95% CI: 0.34–0.85) of vaccine acceptance than those without tests.

Caregiver knowledge about the cause of malaria infection showed a positive association with vaccine acceptance. In the case of correct identification of mosquito bites as the cause of infection with malaria, the odds of willingness to vaccinate were significantly higher (aOR: 3.46, 95% CI: 1.01–11.87) compared to an incorrect identification (e.g. eating certain foods).

### Sierra Leone

Similar to Guinea, in Sierra Leone most caregivers of children who had been ill with fever in the last 30 days resided in rural areas, specifically in the districts of Pujehun (42%, *n* = 239) and Port Loko (38%, *n* = 218) compared to a smaller proportion in the Western Area Urban (20%, *n* = 118), where the capital is situated. 243 (42%) of first respondents living in households with consenting caregivers stated they did not have any formal education, whereas 121 (21%) reported they had finished secondary school or had attained a higher education level. In 271 (47%) of households with consenting caregivers, the first respondent reported their main occupation to be farming (Table 2).

**Table 2:** Sierra Leone: description of study population and vaccine acceptance, results of univariable and multivariable analysis

Characteristics	Households where caregivers consented *N* = 575	Multivariable logistic regression, stepwise selected model based on AIC^a^ *N* = 557^b^ AIC = 535.59, Pseudo-R^2^ (McFadden) = 0.07		
	N (%)	N willing to vaccinate/N total (%)	aOR (95% CI)	p-value
District (*χ*^2^ = 19.41, *p* < 0.001)^e^				
Western Area Urban	118 (20)	105/113 (93)	–	–
Port Loko	218 (38)	170/211 (81)	0.22 (0.08, 0.59)	**0.003**
Pujehun	239 (42)	173/233 (74)	0.17 (0.06, 0.47)	** < 0.001**
Household wealth index (*χ*^2^ = 15.31,* p* = 0.004)^e^				
1st quintile	103 (18)	69/101 (68)	–	–
2nd quintile	139 (24)	107/134 (80)	1.71 (0.93, 3.15)	0.085
3rd quintile	117 (20)	94/114 (83)	1.71 (0.85, 3.42)	0.132
4th quintile	113 (20)	95/110 (86)	1.70 (0.81, 3.58)	0.163
5th quintile	103 (18)	83/98 (85)	0.65 (0.25, 1.66)	0.365
Farming occupation^c^ (*χ*^2^ = 2.65, *p* = 0.104)^e^				
No	304 (53)			
Yes	271 (47)			
Education^c^ (*χ*^2^ = 9.87, *p* = 0.020)^e^				
No formal education	243 (42)	181/236 (77)	–	–
Informal, Koranic or primary school	101 (18)	78/97 (80)	1.29 (0.70, 2.38)	0.421
Intermediate secondary school	109 (19)	83/108 (77)	0.90 (0.51, 1.59)	0.720
Secondary school or higher	121 (21)	106/116 (91)	2.32 (1.05, 5.11)	**0.036**
Missing	0 (0)			
Number of children < 5 years in household (*χ*^2^ = 0.03, *p* = 0.860)^e^				
1	320 (56)			
More than 1	255 (44)			
Number of preventive measures taken against malaria in household (*χ*^2^ = 6.60, *p* = 0.040)^e^				
0–2	264 (46)	202/248 (81)	–	–
3	142 (25)	104/140 (74)	0.55 (0.32, 0.94)	**0.028**
4 or more	169 (29)	142/169 (84)	0.98 (0.57, 1.70)	0.951
Trust in healthcare system^c^ (Fisher’s exact test, *p* = 0.66)^e^				
No or rather no trust	8 (1)			
Some or a lot of trust	555 (97)			
Missing or neutral	12 (2)			
Messages or advertisement about malaria seen during past 6 months^c^ (*χ*^2^ = 0.82, *p* = 0.36)^e^				
No	177 (31)			
Yes	398 (69)			
Caregiver can identify the cause of malaria infection (Fisher’s exact test, *p* = 0.83)^e^				
No	3 (1)			
Partially	93 (16)			
Yes	479 (83)			
Malaria test was performed during child’s febrile illness (*χ*^2^ = 0.34, *p* = 0.56)^e^				
No	42 (7)	30/40 (75)	–	–
Yes	530 (92)	418/517 (81)	1.84 (0.81, 4.15)	0.143
Missing	3 (1)			
Willingness to vaccinate^d^				
Rather unlikely or undecided	112 (19)			
Rather likely	463 (81)			
Missing	0 (0)			

Bolded p-values are considered to be significant

^a^Akaike information criterion

^b^Excluding missing data

^c^Of first respondent

^d^Dependent variable

^e^Result of univariable analysis

#### Malaria vaccine acceptance

In Sierra Leone, caregivers reported a slightly higher vaccine acceptance than in Guinea: 463 (81%) were either likely or very likely to accept a malaria vaccine for their child compared to 112 (19%) who stated they would be very unlikely or unlikely to accept a vaccine, or they were indecisive (Table 2).

#### Factors associated with vaccine acceptance

Results of multivariable analysis to identify factors associated with malaria vaccine acceptance among caregivers in Sierra Leone are displayed in Table 2. Similar to Guinea, an association between the area of residence and malaria vaccine acceptance was found in Sierra Leone: Compared to the Western Area Urban, the odds of willingness to vaccinate were significantly lower in Port Loko (aOR: 0.22; 95% CI: 0.08–0.59) and Pujehun (aOR: 0.17; 95% CI: 0.06–0.47).

In Sierra Leone, the reported education level of the first respondent showed an association with caregiver acceptance of a malaria vaccine: Of those with a first respondent who reported they had finished secondary school or attained a higher educational level, the odds of a likely acceptance of the vaccine were significantly higher (aOR: 2.32; 95% CI: 1.05–5.11) compared to those who reported they did not have any formal education.

Similar to Guinea, results indicate an association between the reported number of preventive measures against malaria taken at the household level and vaccine acceptance. The odds of willingness to vaccinate were significantly lower (aOR: 0.55, 95% CI: 0.32–0.94) for caregivers living in households where three preventive measures were taken, than those households with two or fewer preventive measures. However, odds of acceptance by caregivers in households with four or more preventive measures in place were not significantly different from those living in households applying up to two measures.

## Discussion

This study’s results indicate a high willingness among caregivers of recently-febrile children under 5 years to accept a malaria vaccine for their children in Guinea and Sierra Leone. In both countries, caregivers in rural areas were less in favour of this vaccination. Acceptance also decreased when the reported number of other preventive measures taken against malaria at the household level increased. In Guinea, there was a higher inclination towards vaccination in caregivers who could correctly identify the cause of infection with malaria.

The high proportion of 76% of caregivers indicating acceptance of a malaria vaccination for their children in Guinea and 81% in Sierra Leone mostly aligns with previous findings from other research conducted in sub-Saharan African countries [20, 25]. Although this appears promising, previous work suggests that the stated willingness to get vaccinated to be higher than actual vaccine uptake [39, 40]. Therefore, and in light of a reported prevalence among children aged 0–23 months in Western Africa of about 20% of missed opportunities to get vaccinated with vaccinations they were eligible for, actual malaria vaccine uptake after implementation remains difficult to predict in both countries [41]. Uptake could be further compromised by the fact that the full-vaccination schedule of the malaria vaccine RTS,S/AS01 includes four doses, especially in light of evidence of reduced uptake being associated with the perception of vaccinations for children becoming too many [12, 24]. However, during the first 2 years of the pilot programme in Ghana, Kenya and Malawi, results regarding uptake were positive: At least 70% of targeted children received the first dose, and 62% the third vaccine dose [12].

### Potential challenges for malaria vaccine implementation

Malaria control efforts require a comprehensive approach based on a collection of measures, including prevention, diagnosis, and treatment [8]. So far, vaccination can support elimination as an additional malaria control measure, but not as an alternative to other measures [8]. Effective vaccine implementation within malaria control programmes requires acceptance of both vaccination and other control measures, such as insecticide-treated nets (ITNs) or testing for malaria and subsequent treatment.

A negative association between malaria vaccine acceptance and reported testing for malaria during children’s illnesses was found in Guinea. Further, a negative association was found between malaria vaccine acceptance and increasing reported number of preventive measures taken per household in both Guinea and Sierra Leone, although not significant for the highest number of preventive measures in the latter. This inverse association of vaccine acceptance with other preventive measures and screening behaviour might pose a challenge to successful malaria control. So far, little evidence on associations between malaria vaccine acceptance and the adherence to other control measures exists. Only one study from Nigeria found malaria vaccine hesitancy to be associated with the availability of other preventive measures [15].

Risk perception may positively influence the perceived need for vaccination and thus vaccine acceptance [42]. Assuming that preventive measures are taken due to higher risk perception, intentions to get children vaccinated would be expected to increase accordingly. The results obtained from this study suggest the opposite and therefore may require a more nuanced explanation [43]. Even though risk perception is high, malaria vaccine acceptance might remain low when other preventive measures are available, less invasive, and in use. Interventions like mosquito nets, or preventive antimalarial drug treatment are well-established prevention efforts [43]. They might be better known and a convenient way of protection, while vaccination may be perceived as risky and invasive. This aligns with findings that suggest that the perception that children already receive too many vaccinations presents a barrier to malaria vaccination [24]. Caregivers might view vaccination as less effective than other malaria control measures, potentially leading to lower levels of vaccine acceptance [44]. Similarly, caregivers whose children were tested for malaria when febrile might perceive testing and subsequent treatment as an existing remedy against malaria. They might perceive their child as less vulnerable and consequently in less need for prevention through vaccination.

### Additional considerations for implementation

Many of the associations between the explanatory factors investigated in this study and malaria vaccine acceptance are in line with prior findings from other sub-Saharan African countries [20, 25]. Previous studies of children’s parents and caregivers in sub-Saharan African countries support the identified positive association between malaria vaccine acceptance and educational levels – although only to a limited extent as in the present study only the first respondent’s educational level was assessed [23, 24]. Higher educational attainment and better knowledge about malaria might increase the risk perception of the disease, and consequently vaccine acceptance [29–31, 45].

In Guinea, the inverse association of malaria vaccine acceptance and wealth reflects previous findings [15, 25], yet intuitively contradicts theoretical concepts indicating that cost is a major barrier to vaccination [29]. Wealth, however, might be associated with reduced perceptions of risk of dying of malaria, as richer population groups often have access to better health care and treatment costs pose less of a threat of economic instability, and so they have better health outcomes in general [46–48]. Thus, wealthier participants might perceive less vulnerability and risk associated with malaria and might therefore see less need for vaccination [29]. In contrast to this study’s findings, in the latest Demographic and Health Survey (DHS) conducted in Guinea, the highest wealth quintile exhibited maximum routine childhood vaccination rates [49]. This suggests that even though higher malaria vaccine acceptance was found in the present study among less wealthy participants, actual vaccination uptake might depend on factors related to affordability. However, the DHS results are not adjusted for other factors such as educational levels which might contribute to differences in vaccine uptake. In contrast to the findings from Guinea, the results in Sierra Leone, although not significant, point towards the opposite direction. Higher vaccine acceptance was found among wealth index quintiles two to four compared to quintile one, i.e. caregivers residing in richer households were more accepting of a malaria vaccine. This might suggest that cost-related barriers to malaria vaccine acceptance play a greater role in Sierra Leone than Guinea.

Geographic variation in malaria vaccine acceptance exists in several African countries, though not always as a gradient between urban and rural areas as identified in this study [19, 22, 23]. Generally, regional disparities in acceptance might be due to local variations in access to health care. Access and structural barriers to vaccination are part of several theoretical models aiming to explain vaccine hesitancy or acceptance [29, 45]. The present study’s findings, however, are not likely explained by differences in access, as almost all participants stated that they attend a health facility. Disparities in risk perception might more plausibly underlie the observed geographic variations in malaria vaccine acceptance. Intuitively, one might assume higher risk perception and thus higher acceptance in areas with higher levels of endemicity – an association identified in previous work from Kenya [23]. In the present study however, the highest acceptance was found in urban areas where malaria prevalence in children is lower [7, 10]. Higher risk perception in urban areas could be due to a larger amount of available information about malaria.

## Strengths and limitations

This study adds to the limited evidence on acceptance of malaria vaccination in Guinea and Sierra Leone, and vaccine acceptance in African countries more broadly. Little is known in either Guinea or Sierra Leone about attitudes towards malaria vaccination for children. Knowledge gained from this study might inform targeted strategies to maximize vaccine acceptance after implementation in childhood immunization programs.

However, this study does have some limitations. The cross-sectional study design does not allow for causal conclusions. The generalizability of findings is limited due to the applied sampling strategy, and because only caregivers of children with recent episodes of fever were interviewed. However, there is evidence of LQAS performing similarly to stratified random sampling in the sampled areas, and confidence in the results is further increased by the relatively large sample size in this study [50]. No data on caregiver age and gender was collected, which might bias this study’s results. However, existing evidence of the influence of caregiver age on malaria vaccine acceptance is ambiguous: Studies reported both increased and decreased acceptance with higher age [22, 23], whereas other studies found no association at all [19]. Evidence from previous studies of an effect of the caregiver’s gender on malaria vaccine acceptance was not encountered [23, 27]. Based on prior work conducted in sub-Saharan African countries, it can be assumed that mostly females fulfil child care duties [51–53]. It is uncertain if female vaccine acceptance would translate into actual uptake, as women might not be the ones making decisions about children’ healthcare [24, 49, 54].

As all study participants in Guinea and more than 99.99% in Sierra Leone stated that they had access to health facilities, it was not possible to study the potential association of limited access to healthcare with malaria vaccine acceptance indicated by previous studies [20]. Further, vaccine-related barriers to caregiver acceptance—e.g., safety concerns and cost, which both might play a role in decision-making—were not measured [29, 55]. Theoretical concepts about vaccination and health-seeking behaviour include further dimensions such as collective responsibility, trust, opportunity, compliance and conspiracy [42]. Due to a lack of data relating to these concepts in the present study, they were not discussed. Nevertheless, they might be relevant for malaria vaccination behaviour and future research needs to assess their importance.

## Conclusion

This study’s findings suggest that policy makers may consider geography, wealth, educational level, and knowledge about malaria for upcoming vaccine malaria introduction campaigns in Guinea and Sierra Leone. These findings are particularly important in light of the WHO’s recent recommendation for children at risk to be vaccinated with the novel malaria vaccine RTS,S/AS01 [13]. Future malaria vaccination programmes ought to go hand in hand with sensitization campaigns to enhance knowledge about the need for a comprehensive approach to malaria control, which includes other preventive measures and timely testing and treatment. Further research should focus on causal relationships and address knowledge gaps concerning possible facilitators and barriers related to malaria vaccine uptake and community beliefs.

### Supplementary Information

**Additional file 1:** Variable coding.

**Additional file 2:** Model selection and sensitivity analysis.

**Additional file 3:** Diagnostics and validation.

## Data Availability

The datasets generated and analysed during the current study are not publicly available due to reasons of sensitivity but are available from the corresponding author on reasonable request.

## Abbreviations

AIC: Akaike Information Criterion
aOR: Adjusted odds ratio(s)
CEA-PCMT: Centre d’Excellence Africain pour la Prévention et le Contrôle des Maladies Transmissibles
CI: Confidence interval(s)
COMAHS: College of Medicine and Allied Health Sciences
DHS: Demographic and Health Survey
ECDC: European Centre for Disease Prevention and Control
EPIET: ECDC Fellowship Programme Field Epidemiology Path
LMIC: Low- and middle-income countries
LQAS: Lot quality assurance sampling
PAE: Postgraduate Training for Applied Epidemiology
RKI: Robert Koch-Institute
UGANC: Université Gamal Abdel Nasser de Conakry
WHO: World Health Organization
