# Supplementary material for: Caregiver acceptance of malaria vaccination for children under 5 years of age and associated factors: cross-sectional household survey, Guinea and Sierra Leone, 2022
Source: Malar J. 2023 Nov 20;22:355. doi: 10.1186/s12936-023-04783-0 (PMC10662512; doi:10.1186/s12936-023-04783-0)
Supplement: Supplementary file 1 — Additional file 1: Variable coding. [file 12936_2023_4783_MOESM1_ESM.docx]

## Additional file 1: Variable coding

1. Trust in the healthcare system

The variable denoting the first respondent’s self-reported trust in the healthcare system was collected on a 5-point Likert-scale with the following possible answer options: a lot of trust, some trust, neutral, rather no trust, no trust at all. To compare those with rather no trust and no trust at all to those with some and a lot of trust, the variable was dichotomized and the neutrals were excluded.

|  | Guinea *n*(%) | Sierra Leone *n*(%) |
| --- | --- | --- |
| No trust at all | 2 (0) | 5 (1) |
| Rather no trust | 14 (2) | 3 (1) |
| Neutral | 25 (4) | 12 (2) |
| Some trust | 216 (31) | 194 (34) |
| A lot of trust | 427 (61) | 361 (62) |
| Missing | 18 (2) | 0 (0) |
| Total | 702 (100) | 575 (100) |

|  | Guinea *n*(%) | Sierra Leone *n*(%) |
| --- | --- | --- |
| No trust at all or rather no trust | 16 (2) | 8 (1) |
| Some trust or a lot of trust | 643 (92) | 555 (97) |
| Missing or Neutral | 43 (6) | 12 (2) |
| Total | 702 (100) | 575 (100) |

2. Caregiver’s ability to correctly identify the cause of malaria infection

Participants were asked “What is malaria caused by?” and could choose from the following answer options with the possibility to provide more than one answer:

- Mosquito bites
- Drinking dirty water
- Polluted air
- Dirty environment
- Eating certain foods
- Lack of personal hygiene
- Evil spirits
- Other

Participant answers were grouped into three categories according to their level of correctness:

- Not correct: Participant answers did not include mosquito bites
- Partially correct: Participant answers included mosquito bites and incorrect options
- Correct: Participant answers included only mosquito bites
