# Supplementary material for: Caregiver acceptance of malaria vaccination for children under 5 years of age and associated factors: cross-sectional household survey, Guinea and Sierra Leone, 2022
Source: Malar J. 2023 Nov 20;22:355. doi: 10.1186/s12936-023-04783-0 (PMC10662512; doi:10.1186/s12936-023-04783-0)
Supplement: Supplementary file 2 — Additional file 2: Model selection and sensitivity analysis. [file 12936_2023_4783_MOESM2_ESM.docx]

## Additional file 2: Model selection and sensitivity analysis

Model selection was performed based on AIC using the following R code structure:

*full_model <- glm(vaccine_acceptance ~*

*region/district +*

*education +*

*wealth_index +*

*number_children_under5 +*

*trust_in_healthcare_system +*

*farming_occupation +*

*cause_malaria_identified +*

*malaria_messages +*

*preventive_measures +*

*malaria_test,*

*family = “binomial”,*

*data = dataframe)*

*stepwise_selected_model <- stepAIC(model, direction = “both”)*

1. Guinea

1.1 Model selection

|  | **Model AIC** | **Variables** | **Model AIC when removing/including variable** | **Variable removed** |
| --- | --- | --- | --- | --- |
| 1 | 589.74 | **Included**  Number_children_under5  Education  Malaria_messages  Farming_occupation  Trust_in_healthcare_system  Malaria_test  Preventive_measures  Region  Wealth_index  Cause_malaria_identified | 586.12  587.65  587.78  588.41  590.56  594.80  603.43  611.07  619.69  626.40 | Number_children_under5 |
| 2 | 586.12 | **Included**  Education  Malaria_messages  Farming_occupation  Trust_in healthcare_system  Malaria_test  Preventive_measures  Region  Wealth_index  Cause_malaria_identified  **Not included**  Number_children_under5 | 584.10  584.17  584.69  586.90  591.42  599.52  607.10  615.89  622.69  589.74 | Education |
| 3 | 584.10 | **Included**  Malaria_messages  Farming_occupation  Trust_in_healthcare_system  Malaria_test  Preventive_measures  Region  Wealth_index  Cause_malaria_identified  **Not included**  Education  Number_children_under5 | 582.18  583.05  584.85  589.97  598.70  604.48  615.92  621.50  586.123  587.65 | Malaria_messages |
| 4 | 582.18 | **Included**  Farming_occupation  Trust_healthcare_system  Malaria_test  Preventive_measures  Region  Wealth_index  Cause_malaria_identified  **Not included**  Malaria_messages  Education  Number_children_under5 | 581.12  582.92  588.05  597.24  602.57  613.93  619.63  584.10  584.17  585.72 | Farming_occupation |
| 5 | 581.12 | **Included**  Trust_healthcare_system  Malaria_test  Preventive_measures  Region  Wealth_index  Cause_malaria_identified  **Not included**  Farming_occupation  Education  Malaria_messages  Number_children_under5 | 581.93  586.56  596.03  600.60  616.99  618.45  582.18  582.74  583.05  584.80 | None |

- 1. Sensitivity analysis

The robustness of the findings was assessed by comparing the effect estimates obtained from the reported model to the effect estimates from models after adding variables that had been excluded during the stepwise selection.

Table 1 Stepwise selected model

| **Variables** |  | **OR (95% CI)** | ***p*-value** |
| --- | --- | --- | --- |
| Region | Conakry  Kindia  Mamou | -  0.60 (0.27, 1.33)  0.22 (0.09, 0.50) | -  0.21  0.00 |
| Wealth_index | 1^st^ quintile  2^nd^ quintile  3^rd^ quintile  4^th^ quintile  5^th^ quintile | -  0.49 (0.23, 1.04)  0.87 (0.39, 1.94)  0.34 (0.16, 0.74)  0.10 (0.04, 0.24) | -  0.06  0.73  0.01  0.00 |
| Trust_in_healthcare_system | No or rather no trust  Some or a lot of trust | -  0.29 (0.06, 1.44) | -  0.13 |
| Cause_malaria_identified | No  Partially  Correctly | -  0.56 (0.18, 1.74)  3.46 (1.01, 11.87) | -  0.32  0.05 |
| Preventive_measures | 0–3  4+ | -  0.39 (0.25, 0.62) | -  0.00 |
| Malaria_test | No  Yes | -  0.54 (0.34, 0.85) | -  0.01 |

Table 2 Sensitivity model incl. education

| **Variables** |  | **OR (95% CI)** | ***p*-value** |
| --- | --- | --- | --- |
| Region | Conakry  Kindia  Mamou | -  0.57 (0.25, 1.27)  0.20 (0.08, 0.47) | -  0.17  0.00 |
| Wealth_index | 1^st^ quintile  2^nd^ quintile  3^rd^ quintile  4^th^ quintile  5^th^ quintile | -  0.46 (0.22, 0.99)  0.85 (0.38, 1.90)  0.36 (0.16, 0.77)  0.11 (0.05, 0.24) | -  0.05  0.69  0.01  0.00 |
| Trust_in_healthcare_system | No or rather no trust  Some or a lot of trust | -  0.29 (0.06, 1.42) | -  0.13 |
| Cause_malaria_identified | No  Partially  Correctly | -  0.57 (0.18, 1.76)  3.43 (1.00, 11.79) | -  0.32  0.05 |
| Preventive_measures | 0–3  4+ | -  0.40 (0.26, 0.64) | -  0.00 |
| Malaria_test | No  Yes | -  0.55 (0.35, 0.86) | -  0.01 |
| Education | No formal education  Informal or koranic  Primary or higher | -  0.71 (0.34, 1.49)  0.71 (0.45, 1.13) | -  0.37  0.15 |

Table 3 Sensitivity model incl. Number_children

| **Variables** |  | **OR (95% CI)** | ***p*-value** |
| --- | --- | --- | --- |
| Region | Conakry  Kindia  Mamou | -  0.60 (0.27, 1.34)  0.22 (0.09, 0.50) | -  0.22  0.00 |
| Wealth_index | 1^st^ quintile  2^nd^ quintile  3^rd^ quintile  4^th^ quintile  5^th^ quintile | -  0.49 (0.23, 1.04)  0.88 (0.39, 1.96)  0.35 (0.16, 0.74)  0.10 (0.04, 0.24) | -  0.06  0.75  0.01  0.00 |
| Trust_in_healthcare_system | No or rather no trust  Some or a lot of trust | -  0.29 (0.06, 1.43) | -  0.13 |
| Cause_malaria_identified | No  Partially  Correctly | -  0.57 (0.18, 1.76)  3.51 (1.02, 12.06) | -  0.33  0.05 |
| Preventive_measures | 0–3 | - | - |
|  | 4+ | 0.39 (0.24, 0.61) | 0.00 |
| Malaria_test | No  Yes | -  0.54 (0.35, 0.86) | -  0.01 |
| Number_children_under5 | 1  2  >2 | -  0.97 (0.59, 1.60)  0.87 (0.52, 1.45) | -  0.91  0.58 |

Table 4 Sensitivity model incl. farming occupation

| **Variables** |  | **OR (95% CI)** | ***p*-value** |
| --- | --- | --- | --- |
| Region | Conakry  Kindia  Mamou | -  0.58 (0.26, 1.28)  0.20 (0.09, 0.47) | -  0.18  0.00 |
| Wealth_index | 1^st^ quintile  2^nd^ quintile  3^rd^ quintile  4^th^ quintile  5^th^ quintile | -  0.48 (0.23, 1.04)  0.89 (0.40, 1.98)  0.35 (0.16, 0.77)  0.11 (0.05, 0.26) | -  0.06  0.77  0.01  0.00 |
| Trust_in_healthcare_system | No or rather no trust  Some or a lot of trust | -  0.30 (0.06, 1.46) | -  0.14 |
| Cause_malaria_identified | No  Partially  Correctly | -  0.56 (0.18, 1.75)  3.48 (1.01, 11.98) | -  0.32  0.05 |
| Preventive_measures | 0–3  4+ | -  0.39 (0.25, 0.61) | -  0.00 |
| Malaria_test | No  Yes | -  0.53 (0.34, 0.83) | -  0.01 |
| Farming_occupation | No  Yes | -  1.26 (0.79, 2.00) | -  0.33 |

Table 5 Sensitivity model incl. malaria_messages

| **Variables** |  | **OR (95% CI)** | **p-value** |
| --- | --- | --- | --- |
| Region | Conakry  Kindia  Mamou | -  0.60 (0.27, 1.33)  0.21 (0.09, 0.50) | -  0.21  0.00 |
| Wealth_index | 1^st^ quintile  2^nd^ quintile  3^rd^ quintile  4^th^ quintile  5^th^ quintile | -  0.49 (0.23, 1.04)  0.87 (0.39, 1.93)  0.34 (0.16, 0.73)  0.10 (0.04, 0.24) | -  0.06  0.73  0.01  0.00 |
| Trust_in_healthcare_system | No or rather no trust  Some or a lot of trust | -  0.29 (0.06, 1.44) | -  0.13 |
| Cause_malaria_identified | No  Partially  Correctly | -  0.57 (0.18, 1.78)  3.51 (1.02, 12.10) | -  0.33  0.05 |
| Preventive_measures | 0–3  4+ | -  0.39 (0.25, 0.62) | -  0.00 |
| Malaria_test | No  Yes | -  0.54 (0.34, 0.85) | -  0.01 |
| Malaria_messages | No  Yes | -  0.92 (0.49, 1.72) | -  0.79 |

1. Sierra Leone

2.1 Model selection

|  | **Model AIC** | **Variables** | **Model AIC when removing/including variable** | **Variable removed** |
| --- | --- | --- | --- | --- |
| 1 | 544.68 | **Included**  Cause_malaria_identified  Number_children_under5  Farming_occupation  Trust_in_healthcare_system  Malaria_messages  Malaria_test  Wealth_index  Education  Preventive_measures  District | 541.82  542.76  542.82  543.01  543.77  544.29  544.67  545.17  546.09  553.51 | Cause_malaria_identified |
| 2 | 541.82 | **Included**  Number_children_under5  Farming_occupation  Trust_in_healthcare_system  Malaria_messages  Malaria_test  Wealth_index  Education  Preventive_measures  District  **Not included**  Cause_malaria_identified | 539.90  539.96  540.17  540.95  541.55  542.21  542.37  543.36  550.60  544.68 | Number_children_under5 |
| 3 | 539.90 | **Included**  Farming_occupation  Trust_in_healthcare_system  Malaria_messages  Malaria_test  Wealth_index  Education  Preventive_measures  District  **Not included**  Number_children_under5  Cause_malaria_identified | 538.03  538.36  539.04  539.62  540.32  540.45  541.44  548.77  541.82  542.76 | Farming_occupation |
| 4 | 538.03 | **Included**  Trust_in_healthcare_system  Malaria_messages  Malaria_test  Education  Wealth_index  Preventive_measures  District  **Not included**  Farming_occupation  Number_children_under5  Cause_malaria_identified | 536.38  537.21  537.80  538.45  538.53  539.61  546.83  539.90  539.96  540.89 | Trust_in_healthcare_system |
| 5 | 536.38 | **Included**  Malaria_messages  Malaria_test  Wealth_index  Education  Preventive_measures  District  **Not included**  Trust_in_healthcare_system  Farming_occupation  Number_children_under5  Cause_malaria_identified | 535.59  536.56  536.74  536.86  537.79  545.08  538.03  538.26  538.31  539.22 | Malaria_messages |
| 6 | 535.59 | **Included**  Malaria_test  Education  Wealth_index  Preventive_measures  District  **Not included**  Malaria_messages  Trust_in_healthcare_system  Farming_occupation  Number_children_under5  Cause_malaria_identified | 535.61  535.79  535.81  537.15  544.76  536.38  537.21  537.43  537.51  538.38 | None |

2.2 Sensitivity analysis

The robustness of findings was assessed by comparing effect estimates obtained from the reported model to effect estimates from models after adding variables which were excluded during stepwise selection.

Table 6 Stepwise selected model

| **Variables** |  | **OR (95% CI)** | ***p*-value** |
| --- | --- | --- | --- |
| District | Western Urban  Port Loko  Pujehun | -  0.22 (0.08, 0.59)  0.17 (0.06, 0.47) | -  0.00  0.00 |
| Education | No formal education  Informal, Koranic, primary  Intermediate secondary  Secondary or higher | -  1.29 (0.70, 2.38)  0.90 (0.51, 1.59)  2.32 (1.05, 5.11) | -  0.42  0.72  0.04 |
| Wealth_index | 1^st^ quintile  2^nd^ quintile  3^rd^ quintile  4^th^ quintile  5^th^ quintile | -  1.71 (0.93, 3.15)  1.71 (0.85, 3.42)  1.70 (0.81, 3.58)  0.65 (0.25, 1.66) | -  0.08  0.13  0.16  0.37 |
| Preventive_measures | 0–2  3  4+ | -  0.55 (0.32, 0.94)  0.98 (0.57, 1.70) | -  0.03  0.95 |
| Malaria_test | No  Yes | -  1.84 (0.81, 4.15) | -  0.14 |

Table 7 Sensitivity model incl. cause_malaria_identified

| **Variables** |  | **OR (95% CI)** | **p-value** |
| --- | --- | --- | --- |
| District | Western Urban  Port Loko  Pujehun | -  0.22 (0.08, 0.59)  0.16 (0.06, 0.47) | -  0.00  0.00 |
| Education | No formal education  Informal, Koranic, primary  Intermediate secondary  Secondary or higher | -  1.27 (0.68, 2.36)  0.89 (0.51, 1.57)  2.32 (1.05, 5.10) | -  0.45  0.69  0.04 |
| Wealth_index | 1^st^ quintile  2^nd^ quintile  3^rd^ quintile  4^th^ quintile  5^th^ quintile | -  1.66 (0.90, 3.06)  1.70 (0.85, 3.41)  1.69 (0.80, 3.57)  0.65 (0.25, 1.66) | -  0.10  0.13  0.17  0.36 |
| Preventive_measures | 0–2  3  4+ | -  0.56 (0.32, 0.96)  1.00 (0.57, 1.75) | -  0.03  0.99 |
| Malaria_test | No  Yes | -  1.81 (0.80, 4.10) | -  0.15 |
| Cause_malaria_identified | No  Partially  Correct | -  0.00 (0.00, Inf)  0.00 (0.00, Inf) | -  0.98  0.98 |

Table 8 Sensitivity model incl. trust_in_healthcare_system

| **Variables** |  | **OR (95% CI)** | **p-value** |
| --- | --- | --- | --- |
| District | Western Urban  Port Loko  Pujehun | -  0.21 (0.08, 0.58)  0.16 (0.06, 0.46) | -  0.00  0.00 |
| Education | No formal education  Informal, Koranic, primary  Intermediate secondary  Secondary or higher | -  1.32 (0.71, 2.46)  0.91 (0.52, 1.61)  2.32 (1.05, 5.10) | -  0.38  0.75  0.04 |
| Wealth_index | 1^st^ quintile  2^nd^ quintile  3^rd^ quintile  4^th^ quintile  5^th^ quintile | -  1.74 (0.94, 3.21)  1.72 (0.86, 3.45)  1.71 (0.81, 3.61)  0.65 (0.25, 1.68) | -  0.08  0.13  0.16  0.38 |
| Preventive_measures | 0–2  3  4+ | -  0.54 (0.31, 0.93)  0.97 (0.56, 1.69) | -  0.02  0.92 |
| Malaria_test | No  Yes | -  1.74 (0.75, 4.01) | -  0.19 |
| Trust_in_healthcare_system | No or rather no trust  Some or a lot of trust | -  1.78 (0.30, 10.55) | -  0.53 |

Table 9 Sensitivity model incl. malaria_messages

| **Variables** |  | **OR (95% CI)** | **p-value** |
| --- | --- | --- | --- |
| District | Western Urban  Port Loko  Pujehun | -  0.22 (0.08, 0.60)  0.17 (0.06, 0.48) | -  0.00  0.00 |
| Education | No formal education  Informal, Koranic, primary  Intermediate secondary  Secondary or higher | -  1.27 (0.68, 2.35)  0.88 (0.50, 1.55)  2.34 (1.06, 5.17) | -  0.46  0.65  0.03 |
| Wealth_index | 1^st^ quintile  2^nd^ quintile  3^rd^ quintile  4^th^ quintile  5^th^ quintile | -  1.76 (0.95, 3.24)  1.70 (0.85, 3.42)  1.66 (0.79, 3.51)  0.64 (0.25, 1.65) | -  0.07  0.13  0.18  0.36 |
| Preventive_measures | 0–2  3  4+ | -  0.59 (0.34, 1.01)  1.11 (0.61, 1.99) | -  0.06  0.73 |
| Malaria_test | No  Yes | -  1.89 (0.83, 4.32) | -  0.13 |
| Malaria_messages | No  Yes | -  0.74 (0.43, 1.27) | -  0.28 |

Table 10 Sensitivity model incl. farming_occupation

| **Variables** |  | **OR (95% CI)** | **p-value** |
| --- | --- | --- | --- |
| District | Western Urban  Port Loko  Pujehun | -  0.21 (0.08, 0.58)  0.16 (0.06, 0.46) | -  0.00  0.00 |
| Education | No formal education  Informal, Koranic, primary  Intermediate secondary  Secondary or higher | -  1.31 (0.70, 2.43)  0.93 (0.52, 1.66)  2.41 (1.07, 5.40) | -  0.40  0.80  0.03 |
| Wealth_index | 1^st^ quintile  2^nd^ quintile  3^rd^ quintile  4^th^ quintile  5^th^ quintile | -  1.72 (0.93, 3.16)  1.73 (0.86, 3.48)  1.76 (0.82, 3.78)  0.68 (0.26, 1.78) | -  0.08  0.12  0.15  0.43 |
| Preventive_measures | 0–2  3  4+ | -  0.55 (0.32, 0.94)  0.97 (0.56, 1.69) | -  0.03  0.92 |
| Malaria_test | No  Yes | -  1.82 (0.81, 4.13) | -  0.15 |
| Farming_occupation | No  Yes | -  1.11 (0.66, 1.89) | -  0.69 |

Table 11 Sensitivity model incl. number_children_under5

| **Variables** |  | **OR (95% CI)** | **p-value** |
| --- | --- | --- | --- |
| District | Western Urban  Port Loko  Pujehun | -  0.22 (0.08, 0.60)  0.17 (0.06, 0.47) | -  0.00  0.00 |
| Education | No formal education  Informal, Koranic, primary  Intermediate secondary  Secondary or higher | -  1.28 (0.69, 2.38)  0.90 (0.51, 1.59)  2.32 (1.06, 5.12) | -  0.43  0.72  0.04 |
| Wealth_index | 1^st^ quintile  2^nd^ quintile  3^rd^ quintile  4^th^ quintile  5^th^ quintile | -  1.70 (0.92, 3.13)  1.70 (0.84, 3.40)  1.69 (0.80, 3.57)  0.64 (0.25, 1.65) | -  0.09  0.14  0.17  0.36 |
| Preventive_measures | 0–2  3  4+ | -  0.55 (0.32, 0.94)  0.98 (0.57, 1.71) | -  0.03  0.95 |
| Malaria_test | No  Yes | -  1.84 (0.82, 4.17) | -  0.14 |
| Number_children_under5 | 1  >1 | -  0.94 (0.60, 1.46) | -  0.78 |
