# Supplementary material for: Caregiver acceptance of malaria vaccination for children under 5 years of age and associated factors: cross-sectional household survey, Guinea and Sierra Leone, 2022
Source: Malar J. 2023 Nov 20;22:355. doi: 10.1186/s12936-023-04783-0 (PMC10662512; doi:10.1186/s12936-023-04783-0)
Supplement: Supplementary file 3 — Additional file 3: Diagnostics and validation. [file 12936_2023_4783_MOESM3_ESM.docx]

## Additional file 3: Diagnostics and validation

1. Model accuracy

Model accuracy was calculated using the following R code:

*y_pred <- round(predict(model, dataframe, type = “response”)
A = table(y_pred, outcome_variable)
Accuracy = sum(diag(A))/sum(A)*

Accuracy model Guinea: 80%

Accuracy model Sierra Leone: 80%

1. Goodness of fit
   1. Likelihood-ratio tests

The fit of the stepwise selected models was compared to the fit of the full model by conducting likelihood-ratio tests.

R code:

*lrtest(fullmodel, reducedmodel)*

Guinea:

|  | #Df | LogLik | Df | Chisq | Pr(>Chisq) |
| --- | --- | --- | --- | --- | --- |
| 1  2 | 18  12 | -276.87  -278.56 | -6 | 3.3772 | 0.7602 |

Sierra Leone:

|  | #Df | LogLik | Df | Chisq | Pr(>Chisq) |
| --- | --- | --- | --- | --- | --- |
| 1  2 | 19  13 | -253.34  -254.79 | -6 | 2.9122 | 0.8198 |

- 1. Hosmer-Lemeshow tests

R code:

*performance_hosmer(model, n_bins = 10)*

|  | **Guinea** | **Sierra Leone** |
| --- | --- | --- |
| Chi-squared  df  p-value | 8.221  8  0.412 | 6.010  8  0.646 |

- 1. Pseudo-R2

|  | **Guinea** | **Sierra Leone** |
| --- | --- | --- |
| McFadden  McFaddenAdj  CoxSnell  Nagelkerke  AldrichNelson  LogLIK  LogLIK0 | 0.2350498  0.2020965  0.2299928  0.3427247  0.2072020  -278.5582819  -364.1521764 | 0.07469870  0.02748856  0.07119616  0.11337773  0.06877794  -254.79517693  -275.36455135 |

1. Multicollinearity
   1. Variance inflation factors

Guinea:

|  | **GVIF** | **Df** | **GVIF^(1/(2*Df))** |
| --- | --- | --- | --- |
| Region  Wealth Index  Trust in healthcare system  Cause of malaria infection known  Number of preventive measures  Malaria test performed | 1.825900  1.770371  1.011887  1.148748  1.142111  1.067999 | 2  4  1  2  1  1 | 1.162437  1.074009  1.005926  1.035276  1.068696  1.033440 |

Sierra Leone:

|  | **GVIF** | **Df** | **GVIF^(1/(2*Df))** |
| --- | --- | --- | --- |
| District  Educational level  Wealth index  Number of preventive measures  Malaria test performed | 2.052408  1.213144  2.094999  1.106723  1.062374 | 2  3  4  2  1 | 1.196922  1.032727  1.096852  1.025675  1.030715 |

1. Specification error

The models were assessed for specification error by using link tests:

R code:

*blr_linktest(model)*

Guinea:

|  | **Estimate** | **Std.Err.** | ***z*-value** | **Pr(>\|*z*\|)** |
| --- | --- | --- | --- | --- |
| Intercept  Fit  Fit^2 | 0.00499  1.14179  -0.06327 | 0.13209  0.16268  0.05539 | 0.038  7.018  -1.142 | 0.970  <0.001  0.253 |

Sierra Leone:

|  | **Estimate** | **Std.Err.** | ***z-*value** | **Pr(>\|*z*\|)** |
| --- | --- | --- | --- | --- |
| Intercept  Fit  Fit^2 | -0.2068  1.3503  -0.1168 | 0.3644  0.4848  0.1481 | -0.567  2.785  -0.789 | 0.570  0.005  0.430 |

1. Influential values

Cook's distances and standardized residuals were calculated to identify influential observations.

Figure 3 Model Guinea: Cook's distances incl. highlighting 3 observations with largest Cook's distances


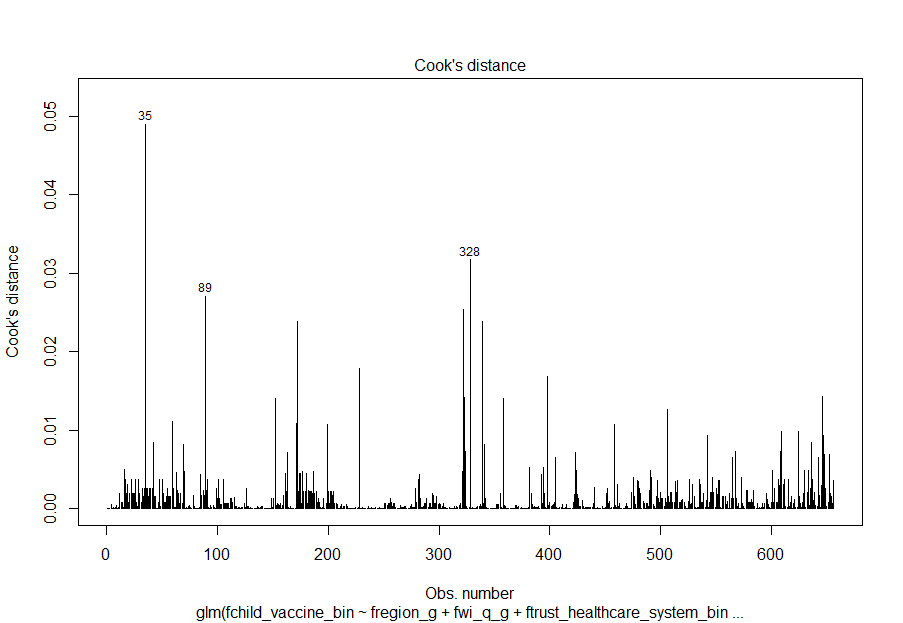


Figure 4 Model Sierra Leone: Cook's distances incl. highlighting 3 observations with largest Cook's distances


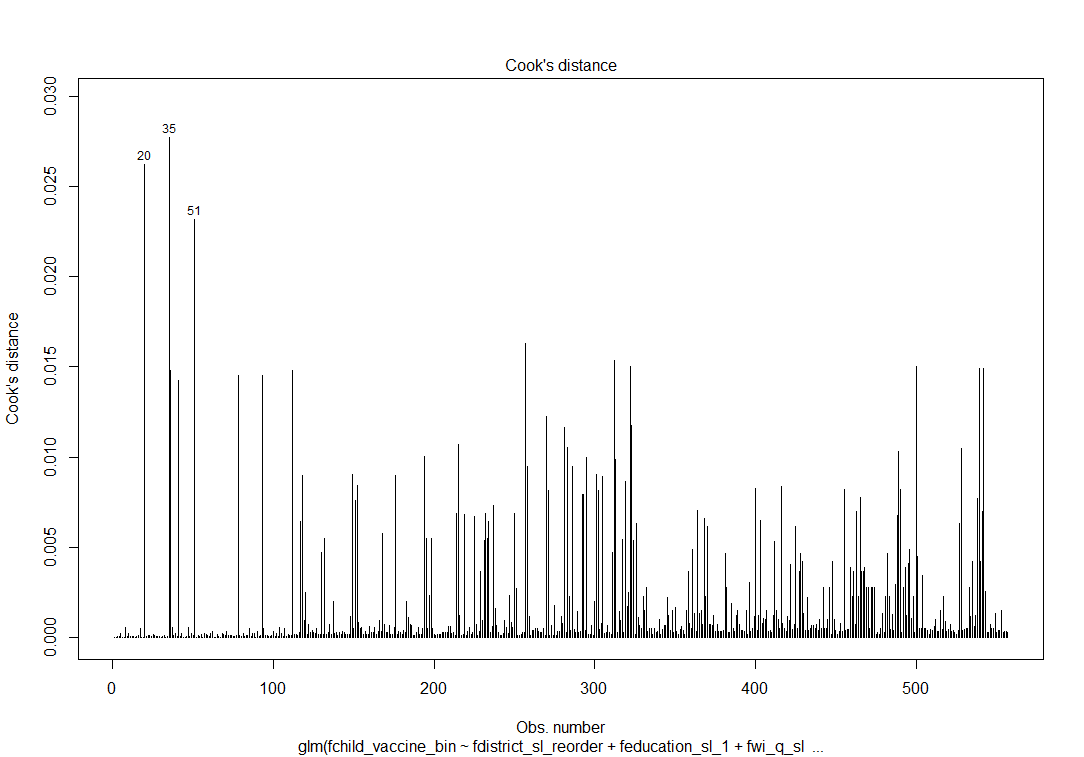


Figure 5 Model Guinea: standardized residuals


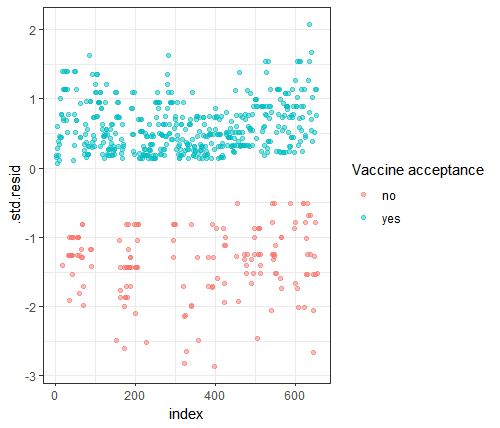


Figure 6 Model Sierra Leone: standardized residuals


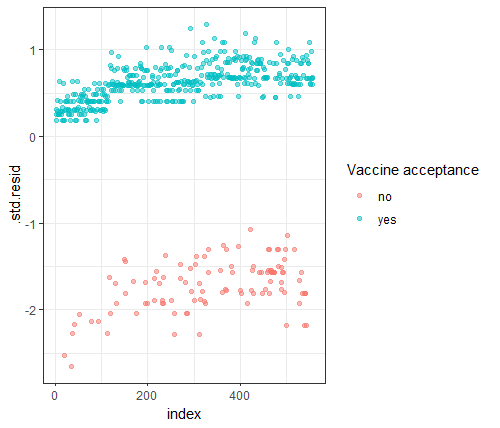


Observations with absolute standardized residuals > 2:

Guinea: 17

Sierra Leone: 18

Observations with absolute standardized residuals > 3:

none
